# Widespread genetic testing controls inherited polycystic kidney disease while avoiding inbreeding in cats

**DOI:** 10.1186/s12864-026-13048-4

**Published:** 2026-06-18

**Authors:** Hisashi Ukawa, Akane Kida, Kai Ataka, Ryo Horie, Yuki Matsumoto

**Affiliations:** 1 Genetic Testing Section, Anicom Pafe Inc, 2-6-3 Chojamachi, Kanagawa, Yokohamashi-Nakaku 231-0033 Japan; 2 Research and Development Section, Anicom Insurance Inc, 8-17-1 Nishi-Shinjuku, Tokyo, Shinjuku-ku 160-0023 Japan; 3 Research and Development Section, Anicom Specialty Medical Institute Inc, 8-17-1 Nishi-Shinjuku, Tokyo, Shinjuku-ku 160-0023 Japan; 4 Data Science Center, Azabu University, 1-17-71 Fuchinobe, Chuo-ku, Sagamihara-shi, Kanagawa Japan

**Keywords:** Cat, Genetic testing, Polycystic kidney disease

## Abstract

**Background:**

Polycystic kidney disease (PKD) is a major hereditary disorder characterized by the formation of cysts in the kidneys of mammals, including humans and cats. It commonly leads to renal failure and is frequently associated with variants in the *PKD1* gene. In cats, a specific *PKD1* variant (chr E3:g.42858112 C > A) is a primary target for direct-to-consumer (DTC) genetic testing aimed at identifying PKD risk. However, its broad impact on the genetic structure of cat populations remains unclear. In this study, we investigated temporal and breed-specific dynamics of feline PKD and the *PKD1* variant using a large-scale dataset comprising 110,325 insured cats and 61,968 genetically tested cats across 14 breeds in Japan.

**Results:**

Our comprehensive analysis of insured cats revealed no clear differences in claim-based detection of cystic kidneys across breeds, sexes, or ages, although notable geographic variations were observed. Among the cats diagnosed with cystic kidneys, 77.8% carried the conventional *PKD1* variant. Exome and whole-genome sequencing of affected cats revealed eight potentially deleterious variants in PKD-related genes, suggesting the involvement of other genetic factors. The carrier frequency of the *PKD1* variant in all 14 breeds decreased by 42.6% when comparing genetic data from 2019, when full-scale genetic testing for kittens began, and 2022. In additional analyses focusing on eight breeds with larger sample sizes, significant reductions in the proportion of heterozygous cats were observed in Scottish Folds, Persians, and Ragamuffins (49.5%, 38.8%, and 57.7% decrease, respectively) between the two time points. In contrast, no statistically significant or suggestive reductions were observed in British Shorthairs, Minuets, or Munchkins. Further genomic analyses in Scottish Folds and Persians indicated no substantial changes in overall genetic structure or inbreeding levels; however, the effective population size of cats harboring the *PKD1* variant declined between these periods.

**Conclusions:**

These findings highlight the role of DTC genetic testing in promoting optimized breeding strategies that contribute to reducing PKD risk while avoiding inbreeding and enhancing feline welfare. To further reduce the number of risk variants, promoting genetic testing and ensuring appropriate breeding practices is necessary, particularly for breeds in which a decrease in *PKD1* variants has not been observed.

**Supplementary Information:**

The online version contains supplementary material available at 10.1186/s12864-026-13048-4.

## Background

Polycystic kidney disease (PKD) is the most frequently inherited kidney disease found in humans [1, 2], cats [3], dogs, and livestock [4]. PKD is either inherited as autosomal dominant PKD (ADPKD) or autosomal recessive types. In cats, ADPKD is one of the most commonly inherited diseases, and a nonsense single-nucleotide variant on chromosome E3 (g.42858112 C > A on the felCat9 assembly) was identified as a causative variant of ADPKD in 2004 [3] (OMIA:000807–9685 [5], the conventional *PKD1* variant). ADPKD is characterized by the development of multiple fluid-filled cysts, primarily within the kidneys and occasionally in the liver or pancreas. These cysts gradually increase in both number and size, thus leading to deterioration of renal function through compression of the surrounding renal parenchyma. The conventional *PKD1* variant is considered to have nearly complete penetrance in cats, causing ADPKD in heterozygous cats and embryonic lethality in homozygous cats [3, 6]. In addition, most cases of feline PKD are caused by the conventional variant (approximately 95% [7]). Because of the late onset of the disease [7 years on average [8], affected cats are typically bred before clinical signs are observed [3].

The allele frequency of the conventional *PKD1* variant has been investigated worldwide, including the USA [3], Mexico [9], UK [10], Switzerland [11], Slovenia [12], Taiwan [13], Turkey [14], Brazil [15] and Japan [16, 17]. Several studies have revealed that Persian and Persian-related cats including Himalayan and Exotic Shorthair have a high prevalence of the variant [12, 15–17]; this variant has also been identified in other cat breeds, such as the Scottish Fold [16] and Sphynx cats [18]. However, researchers in most studies analyzed only several dozens of samples and/or the results were biased as limited records from hospitals were used, primarily secondary care data with relatively high numbers of sick animals [16]. Although samples for genetic studies should be collected from diverse populations and breeds [19], the prevalence of the conventional *PKD1* variant may be biased.

Full-scale direct-to-consumer (DTC) genetic testing for dogs and cats began in Japan around 2017, with testing for puppies and kittens starting in 2019. Such genetic testing for inherited diseases is also being conducted using various genes, including *PKD1*. In dogs, a previous study evaluated changes in the genetic structure of Pembroke Welsh Corgis in Japan [20]. The study reported that the genetic testing of 5,512 dogs for the causative mutation (c.118G > A (p.E40K)) in superoxide dismutase 1 (*SOD1*) showed a significant decrease in prevalence, from 14.5% (95/657) in 2019 to 2.9% (24/820) in 2022, while inbreeding levels remained unchanged. Therefore, DTC genetic testing could also influence the genetic structure of cats. However, its effects on the allele frequency of risk variants and genetic structures remain unclear.

In Japan, a significant proportion of cat owners acquire pet insurance concurrently with purchasing their cats from a pet shop. Currently, approximately 18.6% of pets in Japan are insured. Consequently, integrating this insurance data with genetic testing offers a valuable opportunity to clarify the more general prevalence of the *PKD1* variant using a less biased dataset and to elucidate the relationship between PKD and its genetic determinants. In this study, we aimed to characterize feline cystic kidney diseases, including PKD, and their association with the conventional *PKD1* variant and other genetic factors using a large dataset of 110,325 insured and 61,968 genetically tested cats from 14 different breeds (Table 1).

**Table 1:** Number of insured and genetically tested cats

Breed	Insured		Genetic tested		SNP array-based study*	
	10 years	3 years	All genetic tests	With insurance	2019	2022
Scottish Fold	3,849	31,321	16,451	9,238	20 (8)	20 (9)
American Shorthair	2,698	17,910	10,242	6,229	-	-
Munchkin	1,283	15,272	10,132	5,574	-	-
British Shorthair	491	7,627	6,477	3,670	-	-
Minuet	0	2,655	4,540	2,250	-	-
Ragamuffin	139	2,427	3,928	2,133	-	-
Persian	824	5,587	3,596	1,771	21 (9)	20 (10)
Ragdoll	847	6,564	3,150	1,288	-	-
Exotic Shorthair	123	2,652	2,381	1,156	-	-
Himalayan	90	612	542	261	-	-
Siberian	15	2,072	233	1	-	-
Maine Coon	780	5,174	158	2	-	-
Bengal	332	4,552	88	1	-	-
Russian Blue	1,118	5,900	50	2	-	-
Total	12,589	110,325	61,968	33,576	41 (17)	40 (19)

* Numbers in parentheses for SNP array-based studies indicate the number of heterozygous individuals

## Materials and methods

### Ethics statement

All swab samples were obtained with the written consent of the owners. This study was approved by the Ethics Committee of Anicom Specialty Medical Institute, *Inc.* (*ID*: 2020-06, 2022-02, and 2024-07). All procedures followed institutional and international ethical guidelines, including the Basel Declaration (https://animalresearchtomorrow.org/en).

### Insurance data analysis

In Japan, pedigreed cats are frequently acquired from pet shops and breeders. A subset of owners opted to secure pet insurance at the time of acquisition, primarily for clinically healthy cats aged approximately 8 weeks to 1 year. This private pet insurance program covers veterinary medical expenses, including outpatient visits, hospitalization, and surgical procedures. Pets can typically be newly enrolled up to approximately 7 years and 11 months of age, and policies are generally renewable throughout the lifetime of the animal. A waiting period (e.g., approximately 30 days) is applied after the start of the policy, during which newly diagnosed diseases are not covered. After the initial enrollment, annual renewal procedures are explained to owners who choose to maintain their policies.

We used insured cat data to characterize cystic kidney diseases, including PKD, in a cat population, based on three perspectives: breed difference, sex difference, and age difference. Biometric information and insurance claim data were collected for cats that have been continuously insured to the pet insurance by Anicom Insurance Co., one of the largest pet insurance companies in Japan, from April 1, 2008 to December 31, 2023. We extracted and analyzed data of 12,589 cats for a period of more than 10 years from 14 breeds (Scottish Fold, American Shorthair, Munchkin, British Shorthair, Minuet, Persian, Ragamuffin, Ragdoll, Exotic Shorthair, Himalayan, Siberian, Maine Coon, Bengal, and Russian Blue) (Table 1; Supplementary Fig. 1A). Renal failure is often reported at 3–10 years of age [8], which is why we included data up to 10 years. Individuals diagnosed with PKD or detected renal cysts and for which insurance claims were filed were classified as the cyst-positive group (the cat diagnosed with cystic kidneys). Individuals for which insurance claims were filed for other diseases, including renal diseases other than polycystic kidney disease, were classified as the non-positive group. These records reflect diagnoses reported in insurance claims rather than the true biological onset of disease or diagnoses based on standardized imaging protocols. The age at cystic kidney diagnosis was defined as the age at which PKD or renal cysts were first recorded in insurance claims data.

The differences among breeds and sex were analyzed using Fisher’s exact test with the *fisher.test* function in R version 4.3.3. We estimated 95% confidence intervals (95% CIs) for the data analyses to verify the estimation accuracy using logistic regression analysis with the *glm* function with logit link.

Time-to-event analyses were conducted using the age of each cat as the time scale. Time zero was defined as the age at insurance enrollment (typically ranging from 8 weeks to 1 year). The event of interest was the first insurance claim for PKD or renal cysts, while cats without such records were censored at the end of the observation period. The age-specific risk by claim-based diagnosis age was calculated by dividing the number of individuals who developed the disease at age n by the total number of individuals minus the number of individuals who developed the disease by age n-1. Cumulative incidence was calculated from the age-specific risk estimates based on claim-based diagnosis age. We used logistic regression analysis with the *glm* function in R to investigate changes in the proportion of cyst-positive cats by age. Although no missing data were found for sex or breed, neither variable was included as covariates in the regression model because no clear differences were found in the above analysis.

Using continuous insurance claim data for more than 10 years, the number of affected individuals per prefecture was calculated. Moreover, the number of insured cats was calculated for each prefecture, and this was combined with the previously calculated number of affected individuals to calculate the morbidity rate for each prefecture. The proportion of affected cats by prefecture was plotted using the NipponMap package in R.

To investigate the association between birth year and the proportion of cyst-positive cats, we used 110,325 insured cats aged < 3 years. Here, we have analyzed the data by segmenting them up to 3 years of age to observe the presence or absence of an effect from genetic testing and to understand the recent incidence trends of PKD. The number of cats diagnosed with cystic kidneys was counted by birth years and divided by the total number of cat births in the year. The trend of the proportional change by birth year was assessed using the Cochran-Armitage trend test using the *prop.trend.test* function in R.

To investigate the association between the group (cyst-positive/non-positive) and the variant type of the conventional *PKD1* variant (see methods details below), we used 33,576 insured cats with genetic testing between January 1, 2017, and December 31, 2023 (Supplementary Fig. 1B). In Japan, all cats sold by pet shops and catteries are required by law to be microchipped. In the current study, insurance data were directly linked to the number of microchips in each cat. Cats with genotype data for the conventional *PKD1* variant were counted according to the above criteria. The differences between the groups and variant types were analyzed using Fisher’s exact test via the *fisher.test* function in R.

### Next-generation sequencing data analyses

Two cats diagnosed with cystic kidneys (cases 1 and 2) with no conventional *PKD1* variant and 104 cats from the NCBI sequence read archive (https://www.ncbi.nlm.nih.gov/sra) were targeted with a genome-wide survey to uncover the potential variant associated with PKD and cystic kidneys (Supplementary Table 2). DNA samples from swabs of cases 1 and 2 were obtained. We used the Twist custom exome panel based on high-quality cat genome assembly (AnAms1.0) [21]. Sequence libraries were generated using the manufacturer’s protocol and sequenced using the NovaSeq X Plus platform (Illumina, San Diego, CA, USA).

After fastq generation and quality control using fastp v.0.22.0 [22] with default settings, the sequenced reads were mapped to AnAms1.0 using Illumina’s DRAGEN v 4.0.1. (Illumina), and joint calling using 106 cats were performed using the DRAGEN software. After generating joint-vcf, we annotated genes and the effect of the variant using SnpEff v5.1d [23]. We referred to Kidney Cystic and Ciliopathy Disorders Gene Curation Expert Panel data sets in the ClinGen database, which is a National Institutes of Health-funded resource dedicated to building a central resource (https://clinicalgenome.org/affiliation/40066). First, 121 registered genes in the database were converted to AnAms1.0 gene sets based on its annotation and 97 genes were detected. Second, all variants found in the 97 genes were filtered by three criteria: (1) impact HIGH by SnpEff annotation, (2) minor allele frequency < 0.01, and (3) only found in PKD-affected or cyst-positive cats.

### Animals for genetic testing

We obtained buccal swabs from 61,968 cats from 14 breeds (Scottish Fold, American Shorthair, Munchkin, British Shorthair, Minuet, Persian, Ragamuffin, Ragdoll, Exotic Shorthair, Himalayan, Siberian, Maine Coon, Bengal, and Russian Blue). All cats born between January 1, 2019, and December 31, 2022, were sampled by breeders, owners, and pet stores from around Japan. The breed and birth date data were collected from the owners. Random-bred cats were excluded from the study because of the small number of samples.

### Genotyping of the PKD1 variant

DNA was extracted from oral mucosal tissue using commercial kits (chemagic™ DNA Buccal Swab Kit, PerkinElmer, Waltham, MA, USA; DNAdvance Kit, Beckman Coulter, Brea, CA, USA). Real-time PCR was performed to determine the genotypes of PKD-associated mutations (*PKD1* variant: g.42858112 C > A), specifically wild-type homozygotes (C/C, Wild), heterozygous carriers (C/A, Hetero), and variant homozygotes (A/A, Mutant), as previously reported [3]. A real-time PCR genotyping assay was performed using TaqMan probes with fluorescein amidite (FAM) and 2’-chloro-7’-phenyl-1,4-dichloro-6-carboxyfluorescein (VIC) at one end and a non-fluorescent quencher (NFQ) at the other. The assay was performed using 5.0 µL of FastStart Universal Probe Master (Rox; Roche, Basel, Switzerland), 0.2 µL of Custom TaqMan SNP Genotyping Assay (Thermo Fisher Scientific, Waltham, MA, USA), > 20 ng of DNA, and ultrapure water up to 10 µL. The Custom TaqMan SNP Genotyping Assay included forward (5ʹ-CCTCGGAGCCGCTTCAC-3ʹ) and reverse (5ʹ-TTGGCGCCCAGGAAGAG-3ʹ) primers and two probes, VIC-ACGAGGAGGACGCAACA-NFQ and FAM-CGAGGAGGACTCAACA-NFQ, for the wild-type and mutant alleles, respectively. Real-time PCR was performed on the fast cycle on a QuantStudio7FlexFast96Well Real-time PCR system (Thermo Fisher Scientific) for one cycle at 25 °C for 30 s and 95 °C for 10 min. This was followed by 40 cycles at 95 °C for 15 s, 60 °C for 1 min, and finally one cycle at 25 °C for 30 s. To determine the specificity of this assay, two samples each of wild-type and heterozygous mutant samples were selected randomly, and a water control was run on the genotyping assay. The genotype of each sample was determined by examining the amplification of the reporter dye (FAM or VIC) in an amplification plot. The genome sequence of Felis_catus_9.0 was used as a reference to design the primers and Taqman probes.

Any differences in the proportion of heterozygous cats among the 14 breeds in 2022 were investigated using a chi-square test via *chisq.test* function in R. To investigate the allele frequency trajectory of the *PKD1* variant over 5 years, we used eight breeds with a large sample size (i.e., over 100 cats/breed/year). A logistic regression based on the *glm* function in R was used, with the birth year of each breed as an explanatory variable and the numbers of wild-type and heterozygous individuals as response variables. Differences with Bonferroni-adjusted *P* < 0.05 were considered statistically significant, whereas those with 0.05 ≤ *P* < 0.10 were considered suggestive. The response variable was assumed to follow a binomial distribution, and the link function was a logit. The 95% CI for genotype frequency was calculated using the *binorm.test* function in R.

### SNP array data analyses

We used an SNP genotyping array for cats, the Feline 63 K SNP genotyping array [24] (Illumina), to investigate the effects of genetic testing on the entire genome. We compared genome-wide SNPs from four subpopulations of wild-type and heterozygous *PKD1* risk alleles, all derived from the Scottish Fold and Persian cats born in 2019 and 2022. These two breeds were selected because of the substantial decrease in frequency of the *PKD1* variant during the spread of genetic testing and because sufficiently large sample sizes could be obtained from a broad client base, thereby minimizing sampling bias. Considering the effect of genetic background on array-based analyses, we assessed all cats from the largest clients. We used an SNP genotyping array (Illumina Infinium iSelect 63 K Cat DNA Array) and an Illumina iScan system to detect 63 K SNP genotypes. Genotype coordinates correspond to the genome assembly, Felis_catus_9.0.

Quality control was performed using PLINK version 1.90 [25]. Missing rates for each locus (geno option) and individual cats (mind option) were both < 5%. We did not examine the Hardy–Weinberg equilibrium, as we did not assume random mating for pedigreed cats. Population genetic analyses should not include close relatives [26]; therefore, we conducted robust relationship-based pruning using the king-cutoff option in PLINK version 2.00a2LM (threshold of 0.176) to exclude pairings between monozygotic twins and first-degree relatives. For allele frequency-based quality control, we excluded SNPs with a minor allele frequency < 0.01. We also removed sex chromosome variants and autosomal insertions-deletions because their effects on allele frequency differed from those of autosomal SNPs. The final analysis included 46,821 autosomal SNPs.

After quality control for individual cats, including removal of related animals, 81 cats remained: homozygous wild-type (C/C) cats tested in 2019 (Wild 2019, *n* = 24) and 2022 (Wild 2022, *n* = 21), as well as heterozygous cats tested in 2019 (Hetero 2019, *n* = 17) and 2022 from two breeds (Hetero 2022, *n* = 19; Table 1). The subsequent analyses were performed using this dataset.

### Genetic structure analysis

To clarify the genetic structure of the cat population, we performed a maximum-likelihood ancestry analysis using ADMIXTURE version 1.3 [27] and PCA using PLINK version 1.9 with the default settings. We set the number of populations (K) between 1 and 10 for ADMIXTURE. We used option cv for the cross-validation error calculation to select the optimal *K* value based on the lowest error.

To estimate the genetic relationships within a breed, the neighbor-joining method was implemented in Molecular Evolutionary Genetic Analysis (MEGA) v 10 [28]. The PLINK ped/map format described above was converted to the FASTA format using an in-house script. FASTA files were used as inputs to MEGA. The maximum composite likelihood method was used to compute the evolutionary distance. Tree construction was performed using the neighbor-joining method with 1,000 replicates using the bootstrap test.

### Estimating inbreeding coefficients and heterogeneity

We inferred the inbreeding levels using specimens obtained from genome-wide SNP genotyping. The R package DetectRuns was used to obtain the proportion of times each SNP fell within a run per population, corresponding to locus homozygosity or heterozygosity in the respective population. The following DetectRuns parameters were used: minSNP = 41, maxGap = 106, minLengthBps = 50,000, and minDensity = 1/5000. Statistical tests were performed using the Wilcoxon test in the R software. Statistical significance was set at 0.05. We estimated observed heterozygosity for four groups (two genotype groups from two time points, 2019 and 2022) from two breeds (Scottish Fold and Persian) and their 95% CI using GenoDive v3.0.6 with default settings [29].

### Estimating effective population size

The contemporary *Ne* for each breed was estimated from the genome-wide SNP data using the linkage disequilibrium method in NeEstimator version 2.1 [30]. We performed this analysis for four groups (Wild from 2019 to 2022 and Hetero from 2019 to 2022) of the two breeds, Persian and Scottish Fold.

## Results

### Evaluating PKD and renal cysts trends for insured cats

**Fig. 1:**
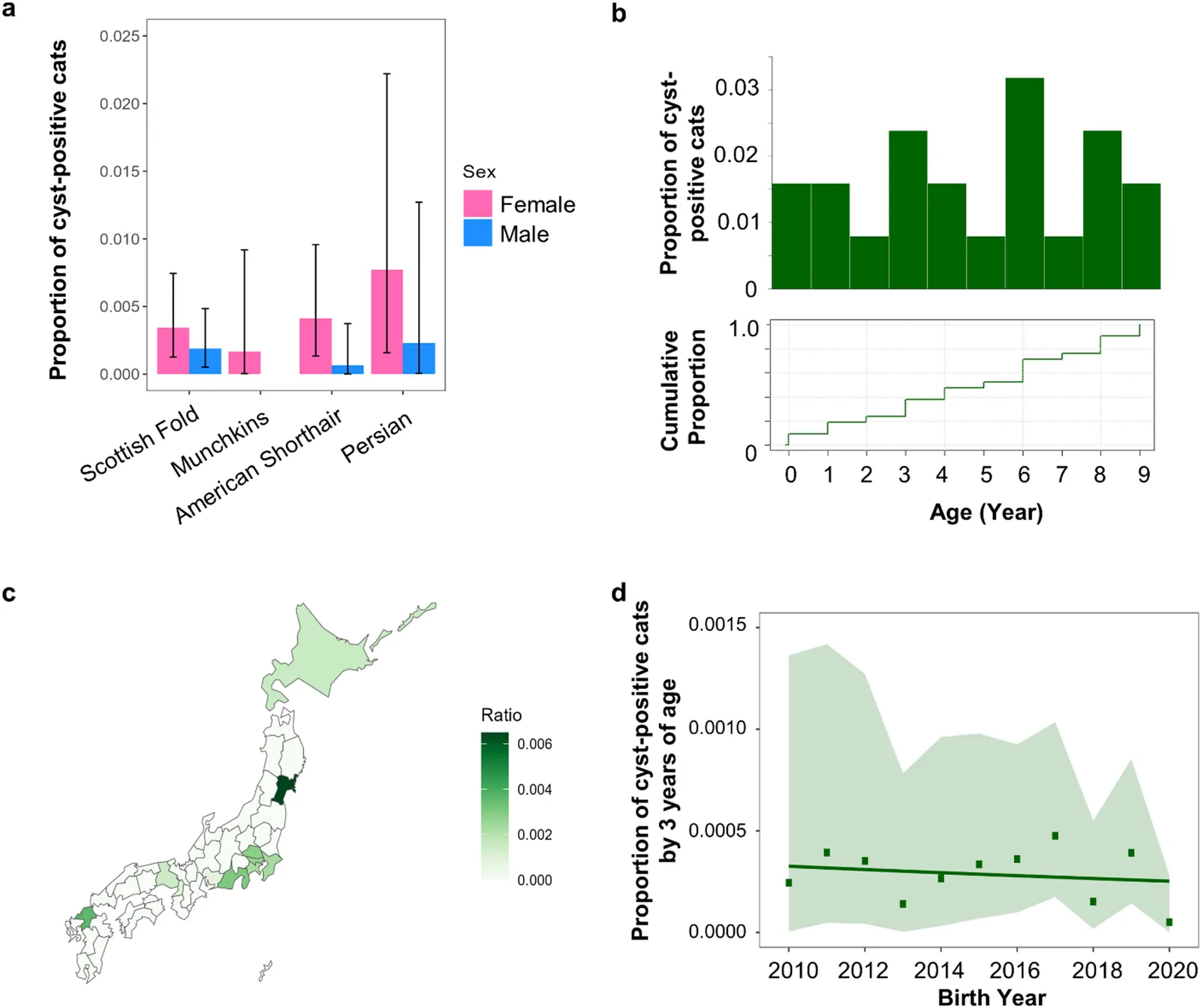
Characterization of cats diagnosed with cystic kidneys based on insured cat data. **a** The proportion of cats diagnosed with cystic kidneys among cats insured for over 10 years, categorized by breed and sex, in four breeds with cyst-positive claims. Error bars indicate 95% CI. **b** Distribution of age of detection of cystic kidneys using cats insured for over 10 years for 4 breeds. Top: frequency based on histogram. Bottom: cumulative proportion by age. **c** Frequency of the cats diagnosed with cystic kidneys across Japan. **d** Frequency of cats diagnosed with cystic kidneys insured for 3 years (14 breeds). Shaded areas indicate 95% CI

First, we confirmed the number of cyst-positive cats, which included cats diagnosed with cystic kidneys and those affected with PKD, according to breed and sex, using a dataset of cats that had been continuously insured for over 10 years (Table 1). We found that cyst-positive cats were identified in only 4 of the 14 breeds tested: Scottish Fold, Munchkin, American Shorthair, and Persian (Fig. 1a and Supplementary Table 1). All four breeds have previously been reported to be affected by PKD [16, 17], and we did not identify significant differences in the status among the breed pairs (Fisher’s exact test: *P* > 0.05). No significant sex differences were observed among cats with renal cysts across the four breeds (Fisher’s exact test: *P* > 0.05), as suggested by previous studies [13, 17, 31]. To further characterize the renal cyst status, we evaluated cats that had been insured for over 10 years, and the rate of cyst-positive cats according to age at first claiming was estimated (Fig. 1b). Of 12,589 cats insured for > 10 years, 21 were diagnosed with cystic kidneys (0.17%; median age, 5 years). No age-related changes were observed between 0 and 10 years of age [logistic regression for age and claim-based diagnosis: 0.026 (SE: 0.076), *P* = 0.73], indicating no association between age and the detection of cystic kidneys. Moreover, the frequency of cats diagnosed with cystic kidneys in Japan differed across the geographic areas (Fig. 1c). To investigate trends in cats diagnosed with cystic kidneys over the past decade and the effect of genetic testing, the proportion of cyst-positive cats (within 3 years of birth) was analyzed. We observed no trend (Cochran-Armitage trend test: X-squared = 0.80, *P* = 0.37, Fig. 1d), indicating that the frequency of claim-based cyst-positive cats has not changed significantly over the past 10 years.

**Fig. 2:**
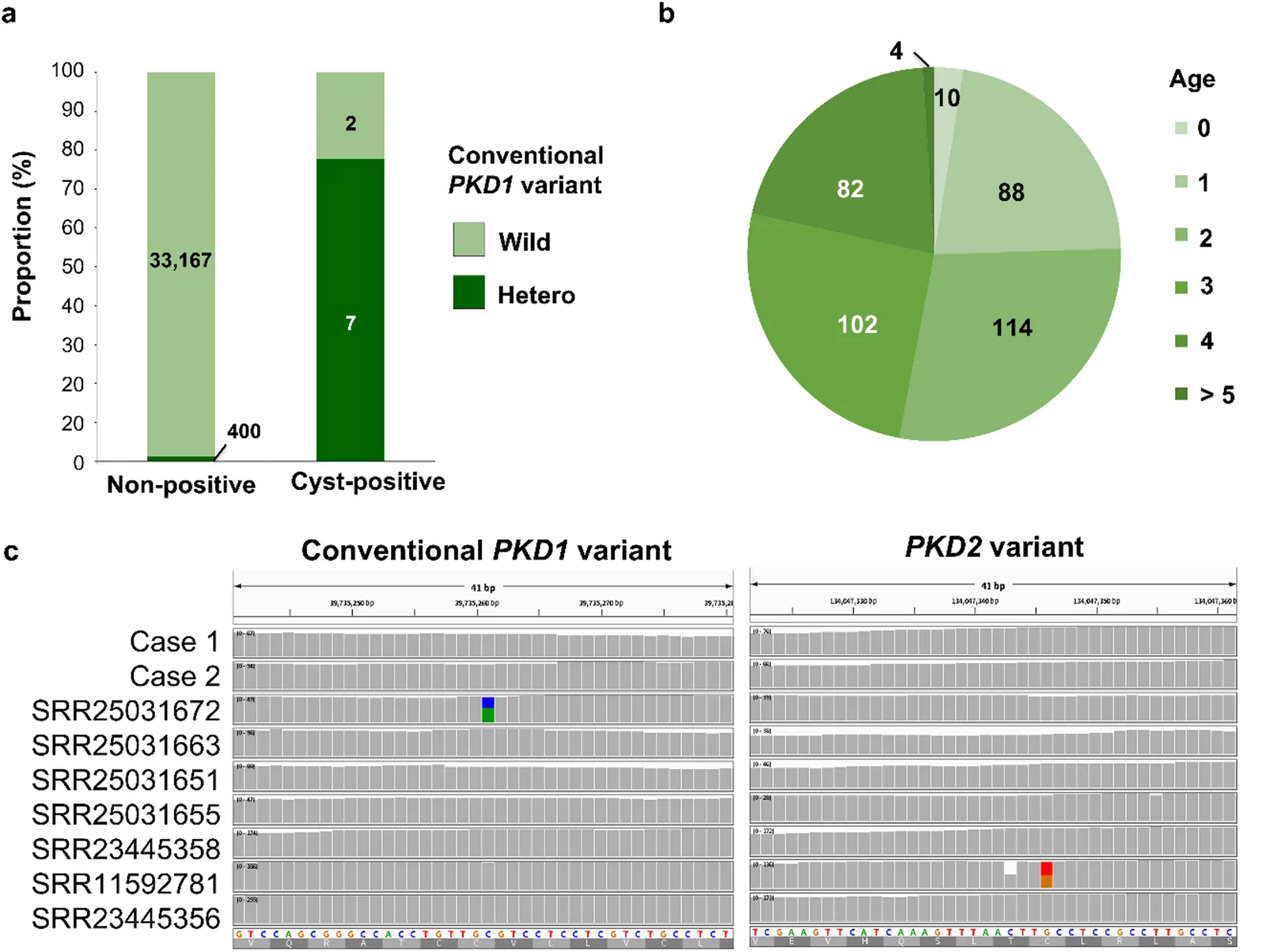
Frequency of the conventional *PKD1* and novel candidate variants associated with PKD. **a** Comparison between conventional *PKD1* genotype (heterozygous vs. wild-type) and cyst-positive status in 14 breeds. **b** Age distribution of cats carrying the conventional *PKD1* variant in the non-positive group (*n* = 400). The number in each segment represents the number of individuals in each age group. **c** IGV view indicating no candidate variants associated with PKD or cystic kidneys in cases 1 and 2, as revealed by whole-genome/exome sequencing

Substantially, we evaluated the association between claims related to cystic kidneys and the *PKD1* variant among 33,576 cats that were insured during the last 3 years. A significant difference was observed between the presence of the conventional *PKD1* variant and the cyst status (Fisher’s exact test; *P* < 1.29e-12, Fig. 2a). We found that 1.2% of non-positive cats (400/33,576 cats) carried the conventional *PKD1* variant. The majority of these cats (314/400; 78.5%) were under three years of age (Fig. 2b), suggesting that these cats may develop PKD in the future. The analysis revealed that seven of the nine cyst-positive cats (77.8%) had the conventional *PKD1* variant while two (22.2%) did not. To identify genetic variants associated with cystic kidneys other than the conventional *PKD1* variant, exome sequencing data for the two cats (cases 1 and 2) and additional open data of whole genome and exome sequencing were analyzed (*n* = 104 including seven PKD-affected cats from web-databases, Supplementary Table 2). Upon searching 97 of 121 genes associated with human PKD, which are orthologs between human and cat genomes, we identified 10 variants, including the conventional *PKD1* variant, as candidates causing PKD (Supplementary Table 3). Three potential variants in *PKD1*, in addition to the conventional *PKD1* variant, were detected in PKD-affected cats in the database. In addition, we identified six high-impact variants in the *IFT80*, *ANKS6*, *RPGRIP1L* and *PKD2* genes, including a *PKD2* frameshift variant that was recently detected [32]. However, no variant was detected in both cyst-positive cases from insured cats (Fig. 2c).

### PKD1 variant trajectories across breeds and years

**Fig. 3:**
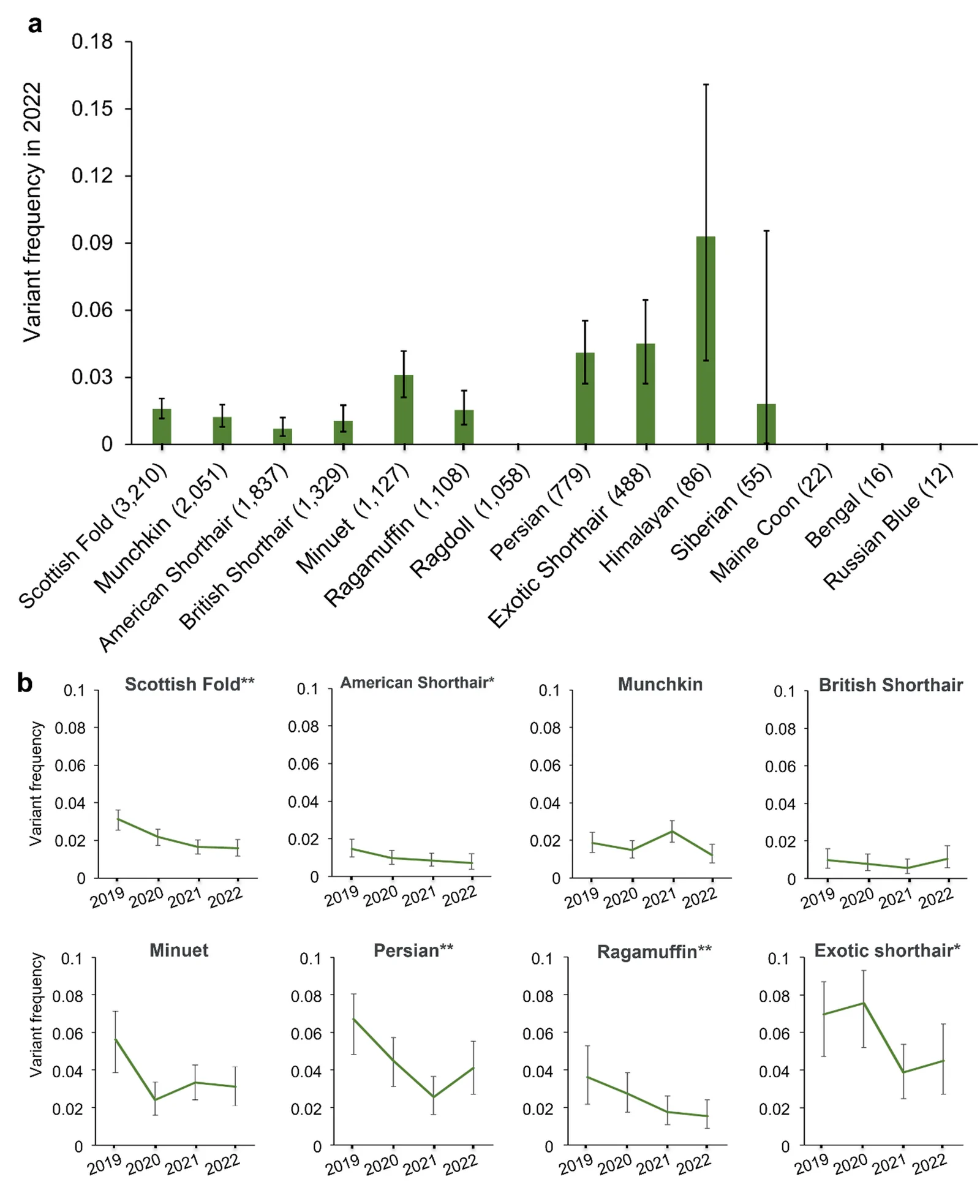
Genotype frequency of heterozygous cats for the *PKD1 *variant. Error bars indicate 95% CIs. **a** Frequency of the *PKD1 *variant in 14 cat breeds born in 2022. The number in parentheses indicates the number of genotyped cats. **b** Trajectory of the proportion of heterozygous cats carrying the *PKD1 *variant in eight breeds with larger annual sample sizes (n > 100). Trends were analyzed using logistic regression. * and ** adjacent to breed names indicate suggestive and significant decreases, respectively

To clarify the frequency of the conventional *PKD1* variant in cats and evaluate the effect of widespread DTC genetic testing on variant prevalence and inbreeding over time, we analyzed a large dataset of 61,968 cats from 14 breeds (Table 1). Real-time PCR was performed to determine the cat genotypes. In 2022, the proportion of individuals heterozygous for the *PKD1* variant differed significantly among the examined breeds, as determined using the chi-square test (X-squared = 133.05, df = 13, p-value < 2.2e-16). No cats were found to be heterozygous for the conventional *PKD1* variant among Ragdoll, Maine Coon, Bengal, and Russian Blue; however, the *PKD1* variant was found in the eight remaining breeds (Fig. 3a and Supplementary Table 4). For the eight breeds, the conventional *PKD1* variant frequency (percentage of heterozygous individuals) ranged between 0.99% (British Shorthair) and 14.07% (Himalayan) in 2019 and between 0.71% (American Shorthair) and 9.30% (Himalayan) in 2022. We identified no cats with homozygous *PKD1* variants in the datasets, consistent with previous reports suggesting that homozygosity for this variant is embryonic lethal [3, 7]. Our findings indicate a 42.6% decline in the frequency of the *PKD1* variant across all breeds when comparing data from 2019, when widespread genetic testing in kittens began, with data from 2022. (Supplementary Table 4).

Next, to accurately investigate the decrease in carrier frequency associated with genetic testing, we performed logistic regression analysis on 8 of the 14 breeds examined, which had larger datasets (over 100 cats/breed/year). Among the eight breeds analyzed using the logistic regression model, the proportion of cats carrying the conventional *PKD1* variant decreased significantly in three breeds of cats born between 2019 and 2022 (Scottish Fold, Persian, and Ragamuffin) and showed suggestive decreases in two breeds (American Shorthair and Exotic Shorthair; Fig. 3b and Supplementary Table 5). No significant or suggestive changes were observed in the remaining three breeds (Munchkin, British Shorthair, and Minuet).

### Effect of genetic testing on population genetic structure

**Fig. 4:**
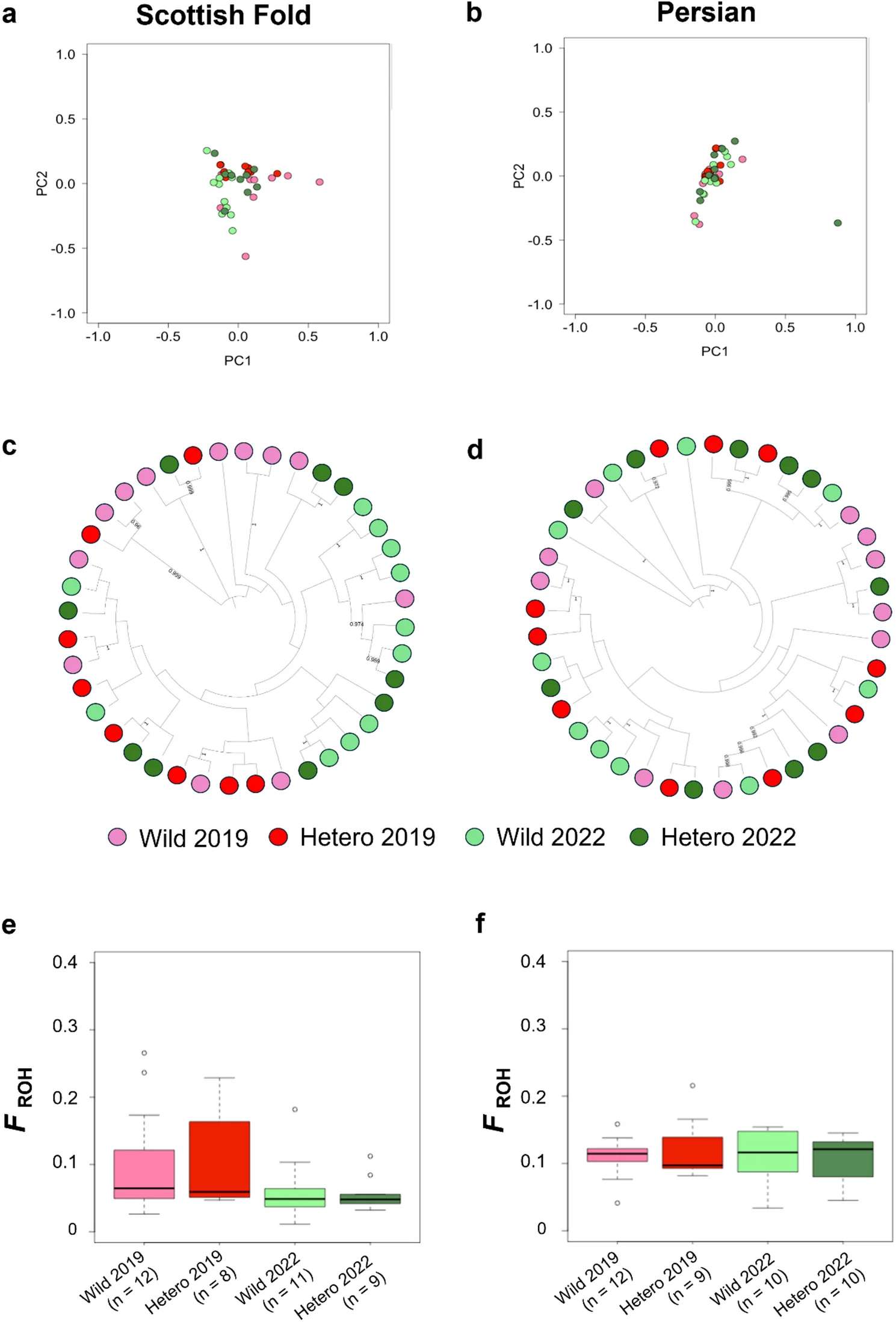
Genetic relationships and inbreeding levels of two breeds. **a**, **b** Principal component analysis (**a**: Scottish Fold, **b**: Persian). **c**, **d** Phylogenetic tree based on the neighbor-joining method (c: Scottish Fold, d: Persian). The percentage of replicate trees in which the associated taxa clustered together in the bootstrap test (1,000 replicates) is shown next to the branches. Only over 95% replicates are shown. Inbreeding coefficient based on runs of homozygosity (**e**: Scottish Fold; **f**: Persian). No significant differences was observed in any comparison

Genome-wide single-nucleotide polymorphisms (SNPs) were assessed in 81 cats to clarify the genetic structure (Table 1), genetic diversity, and effective population size at two time points representing the early and widespread phases of genetic testing: 2019 and 2022, respectively. We analyzed the genetic structure through clustering using ADMIXTURE and principal component analysis (PCA) for two breeds: Scottish Fold and Persian. These two breeds were selected because of the significant decrease in the *PKD1* variant following the spread of genetic testing and because relatively large sample sizes could be included to reduce sampling bias. The clustering results showed that the cross-validation error was the lowest when *K* = 1 in both breeds, indicating no detectable structure in the compared populations. Similarly, PCA revealed no clear separation according to genotype group or birth year (Fig. 4a, b). Furthermore, no clear clusters were identified in the neighbor-joining tree (Fig. 4c, d). We compared the inbreeding coefficients between the groups based on runs of homozygosity (*F*_ROH_; Fig. 4e, f). The mean *F*_ROH_ of the Scottish Fold breed was 0.10 ± 0.081 (SD), 0.10 ± 0.078, 0.06 ± 0.046, and 0.06 ± 0.026 for the wild-type cats born in 2019 (Wild 2019), cats with the conventional *PKD1* variant born in 2019 (Hetero 2019), wild-type cats (Wild 2022), and cats with the conventional *PKD1* variant born in 2022 (Hetero 2022), respectively. The mean *F*_ROH_ of the Persian breed was 0.11 ± 0.029 (SD), 0.12 ± 0.045, 0.11 ± 0.038, and 0.11 ± 0.034 for the Wild 2019, Hetero 2019, Wild 2022, and Hetero 2022 groups, respectively. Overall, no significant differences in *F*_ROH_ were detected (Fig. 4e, f, Wilcoxon test, *P* > 0.05). In addition, the observed heterozygosity of the two breeds was computed for the four groups to investigate genetic diversity. No significant differences were observed among the four groups of either breed (Supplementary Table 6).

**Fig. 5:**
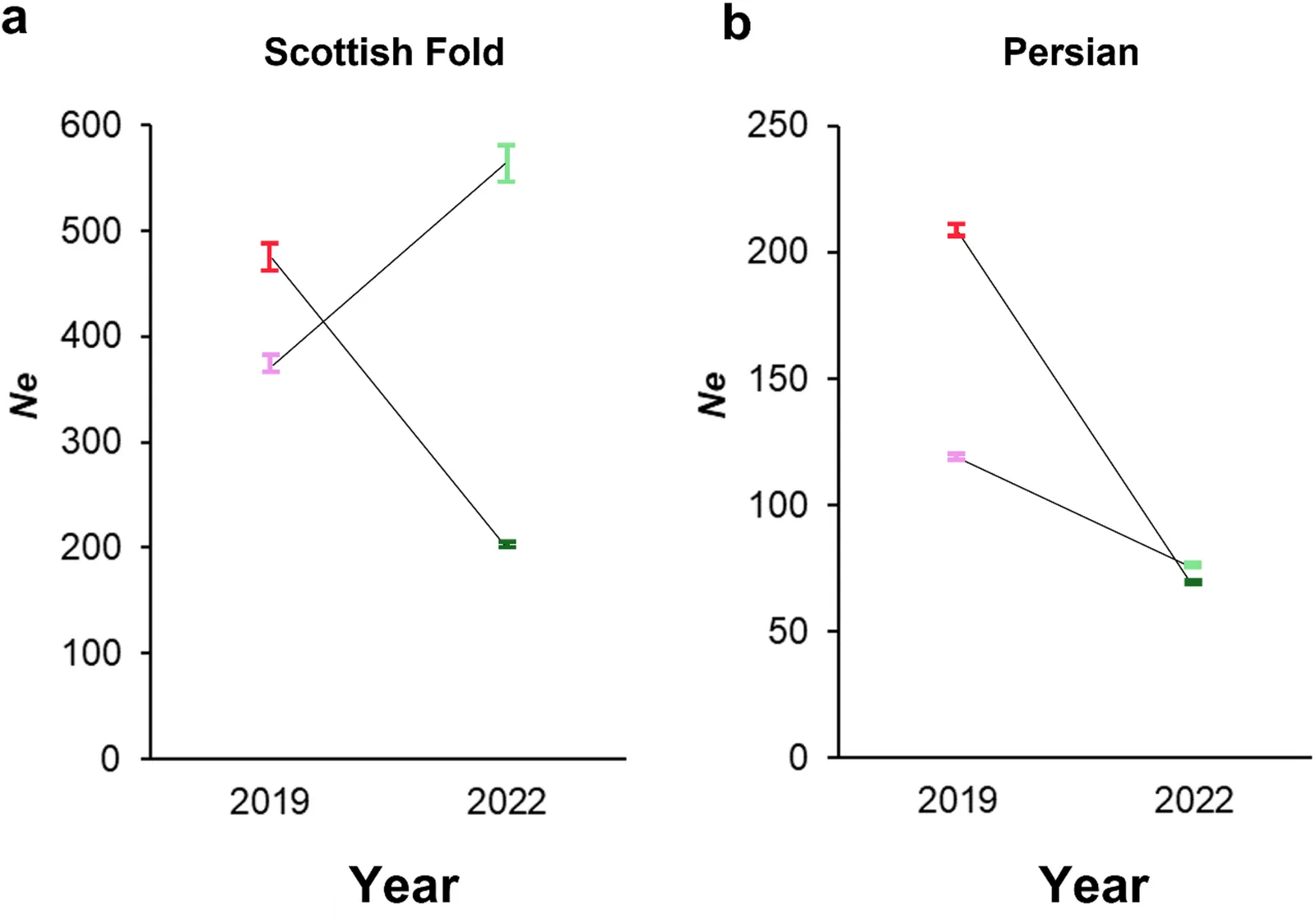
Effective population size (*Ne*) estimated using the genome-wide SNP data. **a** Scottish Fold, **b** Persian. Pink, red, light green, and dark green indicate Wild 2019, Hetero 2019, Wild 2022, and Hetero 2022, respectively. Error bars indicate 95% CIs for *Ne*. CI, confidence interval

To estimate the potential number of cats bred, we used the linkage disequilibrium method to determine the contemporary effective population size, *Ne* (Fig. 5). In the Scottish Fold breed, we computed the contemporary *Ne* of the Wild 2019 group at 375.3 (95% confidence interval [CI]: 367.3–383.7), which was lower than that of the Wild 2022 group at 563.7 (95% CI: 546–582.4), whereas the *Ne* of the Hetero 2019 group was 475 (95% CI: 462.1–488.6), which was higher than that of the Hetero 2022 group at 202.5 (95% CI: 200.1–204.9). These results indicate a marked reduction in the effective population size of cats carrying the *PKD1* variant after the widespread adoption of genetic testing in the Scottish Fold population. In contrast, in the Persian breed, the *Ne* of the Wild 2019 group was 119.2 (95% CI: 118.4–120), which was higher than that of the Wild 2022 group at 76.4 (95% CI: 76.1–76.7), whereas the *Ne* of the Hetero 2019 group was 208.7 (95% CI: 206.3–211.1), which was higher than that of the Hetero 2022 group at 70.1 (95% CI: 69.8–70.4). Estimation of the effective population size suggested that the decline in *Ne* was greater in the population with the conventional *PKD1* variant.

## Discussion

Genetic testing for disease-causing mutations in companion animals is increasingly being performed by veterinarians for diagnosis, by breeders for reducing the incidence of inherited diseases, and by pet owners for determining the genetic background of their pets [33]. Previous reports have identified the conventional *PKD1* variant in at least 11 cat breeds—including Persian, Exotic Shorthair, Himalayan, American Shorthair, Maine Coon, Munchkin, Ragdoll, Scottish Fold, Scottish Straight, Siberian, and Sphynx—as well as in mixed-breed cats. Our study identified this variant in three additional breeds: British Shorthair, Minuet, and Ragamuffin. The prevalence of the *PKD1* variant has been investigated over the past few decades, with the Persian breed being associated with a relatively high prevalence in Japan [46% [17] and 10.81% [16]. In contrast, our results indicate a low prevalence of the variant in the Persian breed (4.11–6.72%). Previous studies used cats that visited university veterinary teaching hospitals as the sampling pool; therefore, the collection was likely to be biased toward unhealthy cats. We considered that collecting samples from a specific prefecture may bias the prevalence of PKD. Therefore, using a more general population for data collection and sampling from a wide geographic area are important to accurately clarify the prevalence of PKD with less biased epidemiological data.

Population genetic studies of cats have shown that the genetic structure varies among countries [34]; therefore, the frequency of the *PKD1* variant may vary not only among breeds and birth years but also among countries. A previous study reported the conventional *PKD1* variant in Maine Coons and Ragdolls. However, our results have suggested that the conventional *PKD1* variant is largely absent in Japanese Maine Coons (0 of 158 cats in our study; 0 of 31 cats reported in previous study [16]; 2 of 4 cats reported in previous study [17]), and no Ragdoll cats harbored the *PKD1* variant among the 3,101 cats tested, indicating limited value of genetic testing in Japanese population. Furthermore, the *PKD1* variant was not found in Ragdoll cats in Japan [16] and Belgium [35]. To date, no *PKD1* variant has been confirmed in Ragdoll cats outside of the USA [3, 36]. In contrast, we observed a higher variant frequency in the Himalayan breed in Japan. In Mexico and Taiwan, the *PKD1* variant has not been identified in any of the Himalayan cats; however, only a small number of cats have been tested [9, 13]. Moreover, previous studies have identified *PKD1* variants in randomly bred cats, in addition to inbred cats [10, 16, 17]. Considering the existence of this variant in diverse cat breeds and randomly bred cats in several countries, the origin of the *PKD1* variant remains unknown. Therefore, future studies are necessary to identify the genetic origin of this variant to reveal the differences in prevalence across breeds and countries.

The frequency of the *PKD1* variant in the Scottish Fold, Persian, and Ragamuffin breeds has decreased significantly after the widespread adoption of genetic testing, when compared to before. This indicates that genetic testing enabled the exclusion of mutant carriers from breeding programs, leading to the utilization of PKD-low-risk non-*PKD1* variant cats for breeding. Supporting this, array-based estimations of *Ne* in Scottish Folds and Persians suggested that the reduction in *Ne* was greater in populations with the conventional *PKD1* variant. Furthermore, no differences were observed in the degree of inbreeding between the two breeds before and after the expansion of genetic testing. This suggests that breeders may have proceeded with breeding plans by mating individuals without the *PKD1* variant while simultaneously avoiding inbreeding. These findings suggest that, at least in the Scottish Fold and Persian breeds, appropriate breeding practices were implemented by not using cats with the conventional *PKD1* variant for mating and actively avoiding inbreeding, as previously reported in dogs [20].

Our results suggest that genetic testing can substantially reduce the frequency of the conventional *PKD1* variant, although its effectiveness may vary among breeds. We identified no critical factor that explains the observed differences; however, the intent of cat breeders and/or prevalence of genetic testing depending on the cat breeds are potential factors. The conventional *PKD1* variant should be eradicated via careful breeding with genetic monitoring to avoid the spread of the variant in all breeds with the existence of the variant, especially for breeds showing no changes, as well as Persian-related breeds, such as Minuet, and high-prevalence breeds, such as Himalayan.

Cyst-positive claims were first recorded at approximately 5 years of age, somewhat earlier than the clinically diagnosed onset of approximately 7 years reported previously [8], which may reflect the claim-based nature of detection in this study. A relatively small percentage (approximately 5% [7] to 22.2% in this study) of cats develop PKD in the absence of the *PKD1* variant; therefore, most cases of PKD in cats are caused by the *PKD1* variant. Our insurance data analysis did not indicate a clear decrease in PKD prevalence during the study period. However, considering the start of widespread DTC genetic testing in Japan, the corresponding decrease in the prevalence of the *PKD1* variant may become apparent in the late 2020s. Most inbred cats are bred by humans, and some genetic variants, including the conventional *PKD1* variant, are considered to have nearly complete penetrance in cats [3, 6]; therefore, to improve animal welfare, breeders should control the risk of such inherited diseases using genetic testing and desired breeding practices based on the results [17, 37].

Several studies have reported cats without the conventional *PKD1* variant, revealing PKD-positive histological evidence and indicating the existence of other variants causing similar pathologies [7, 10, 16]. In fact, we have newly identified eight candidate variants that may cause PKD by searching through in silico analyses, in addition to the conventional *PKD1* and *PKD2* variants reported in previous studies. Although detailed clinical records have not been obtained for such data deposited in databases, and the association between these variants and pathology remains unclear, a causative variant of PKD other than the conventional *PKD1* variant may exist. However, no variant was detected in insured cats in either case, suggesting that other unknown genetic factors such as cis-regulatory elements and/or epigenetic factors might be associated with PKD onset [7]. Therefore, further research, including more comprehensive datasets obtained by pan-genome approaches, is required to genetically and epigenetically characterize feline PKD.

Insurance data can reveal serious medical conditions that may pose a significant burden on population health, even if they are not evident in secondary hospital caseloads. Furthermore, as demonstrated in this study, insurance data have the potential to visualize regional differences [38]. However, medical insurance information is an aggregate of the diagnoses made by individual veterinarians. These data may be subject to bias because of variations in the equipment available at each facility, differences in the veterinarians’ clinical experience and skills, and the possibility that veterinarians may refer to different protocols or guidelines. Especially the timing observed in this study reflects claim-based diagnosis rather than the true biological onset of the disease, which is an inherent limitation of studies using insurance claims data. The outcomes analyzed in this study do not represent the true biological onset of PKD or diagnoses confirmed through standardized imaging protocols, but rather reflect diagnostic and detection patterns recorded within the insurance claims system. Because the dataset was derived from privately insured cats, selection bias may exist due to differences in insurance enrollment rates among breeds or owners. Furthermore, the 10-year continuous insurance dataset was restricted to cats that remained alive and maintained their policies throughout the entire follow-up period, which may introduce survival bias and limit the generalizability of the findings to the broader cat population. Although these biases and limitations exist, in the current study, the frequency of the *PKD1* variant in the cat group diagnosed with kidney cysts was 77.8%, which aligns with findings from previous secondary hospital studies in the Japanese population: 79.3% (23/29) [16] and 79.2% (126/159) [17]. This suggests that for this specific condition, the clinical data from the insurance information are consistent with the secondary hospital data. This consistency might be influenced by the relatively easy diagnosis of renal cysts and the high mutation frequency and penetrance within the population.

## Conclusions

Overall, our findings support the role of DTC genetic testing in promoting optimized breeding strategies that contribute to reduced PKD prevalence and improved feline welfare. To further reduce the number of risk variants, promoting genetic testing and ensuring appropriate breeding practices is necessary, especially for breeds for which a decrease in conventional *PKD1* variants has not been observed. Although our findings suggest that most PKD cases in insured cats possess the conventional *PKD1* variant, unknown genetic factors may also be involved. Furthermore, the genetic structure of cats may differ among countries [34]. More genetic evidence is needed to understand the onset of PKD caused by unknown factors, as well as nationwide and worldwide investigations to identify other genetic variants associated with PKD in cats.

## Supplementary Information

Supplementary Material 1. Supplementary Table 3. Candidate variants reported in PKD-affected/cyst-positive cats based on whole-genome and exome sequencing

Supplementary Material 2. Supplementary Table 2. Whole-genome and exome sequencing to detect novel variants associated with PKD

Supplementary Material 3. Supplementary Figure 1. Flowchart of study population selection and outcome classification.A. The study population criteria, which corresponding to Figure 1. Age at diagnosis was defined as the age at which PKD-related descriptions first appeared in the insurance claims data. B: The study population criteria, which corresponding to Figure 2a-b

Supplementary Material 4. Supplementary Table 6. Comparison of observed heterozygosity

Supplementary Material 5. Supplementary Table 5. Logistic regression statistics for fluctuations in PKD1 variant frequency

Supplementary Material 6. Supplementary Table 4. Number of cats used for allele frequency analysis of conventional PKD1 variants

Supplementary Material 7. Supplementary Table 1. Number of cats insured for >10 years and renal cyst status

## Data Availability

The sequencing data generated in this study are available in the DDBJ Sequence Read Archive under BioProject accession number PRJDB19172. The data can also be accessed through the NCBI SRA (https://www.ncbi.nlm.nih.gov/bioproject/PRJDB19172; exome sequencing data) and the archives of the DRYAD database (https://datadryad.org/dataset/10.5061/dryad.6hdr7sr82; SNP data).
