# Supplementary material for: Widespread genetic testing controls inherited polycystic kidney disease while avoiding inbreeding in cats
Source: BMC Genomics. 2026 Jun 18;27:716. doi: 10.1186/s12864-026-13048-4 (PMC13520345; doi:10.1186/s12864-026-13048-4)
Supplement: Supplementary file 1 [file 12864_2026_13048_MOESM1_ESM.docx]

Supplementary Table 3. Candidate variants reported in PKD-affected/cyst-positive cats based on whole-genome and exome sequencing

| Gene | Chr | Pos | Ref | Alt | Variant type | Case 1 | Case 2 | |
| --- | --- | --- | --- | --- | --- | --- | --- | --- |
| *PKD2* | AnAms1.0_B1 | 134047342 | AC | A* | Frameshift variant | 0/0 | 0/0 | |
| *IFT80* | AnAms1.0_C2 | 106902743 | C | T | Stop gained variant | 0/0 | 0/0 | |
| *ANKS6* | AnAms1.0_D4 | 62462729 | A | C | Splice donor variant & intron variant | 0/0 | 0/0 | |
| *RPGRIP1L* | AnAms1.0_E2 | 31949652 | CT | C | Splice acceptor variant & intron variant | 0/0 | 0/0 | |
|  | AnAms1.0_E2 | 32006570 | CCT | C | Frameshift variant | 0/0 | 0/0 | |
|  | AnAms1.0_E2 | 32009427 | G | T | Stop gained | 0/0 | 0/0 | |
| *PKD1* | AnAms1.0_E3 | 39722018 | GGCCACACCCA | G | Frameshift variant | 0/0 | 0/0 | |
|  | AnAms1.0_E3 | 39735261 | C | A** | Stop gained | 0/0 | 0/0 | |
|  | AnAms1.0_E3 | 39740363 | GAA | G | Stop gained | 0/0 | 0/0 | |
|  | AnAms1.0_E3 | 39740367 | GGTCAAGA | G | Stop gained | 0/0 | 0/0 | |
|  | | | | | |  |  | |
|  | | | | | |  |  | |
| SRR11592781 | SRR23445356 | SRR23445358 | SRR25031651 | SRR25031655 | SRR25031663 | SRR25031672 |  | |
| 0/1 | 0/0 | 0/0 | 0/0 | 0/0 | 0/0 | 0/0 |  | |
| 0/1 | 0/0 | 0/0 | 0/0 | 0/0 | 0/0 | 0/0 |  | |
| 0/0 | 0/0 | 0/0 | 0/0 | 0/0 | 0/0 | 0/1 |  | |
| 0/0 | 0/0 | 0/0 | 0/1 | 0/1 | 0/0 | 0/0 |  | |
| 0/0 | 0/0 | 0/0 | 0/1 | 0/1 | 0/0 | 0/0 |  | |
| 0/0 | 0/0 | 0/0 | 0/1 | 0/1 | 0/0 | 0/0 |  | |
| 0/0 | 0/0 | 0/1 | 0/0 | 0/0 | 0/0 | 0/0 |  | |
| 0/0 | 0/0 | 0/0 | 0/0 | 0/0 | 0/0 | 0/1 |  | |
| 0/0 | 0/1 | 0/0 | 0/0 | 0/0 | 0/0 | 0/0 |  | |
| 0/0 | 0/1 | 0/0 | 0/0 | 0/0 | 0/0 | 0/0 |  | |
| Each genotype was wild-type homozygous (0/0), heterozygous (0/1). * Variant previously reported in a limited colony of Siberian Cat (Rodney et al., 2021). ** Conventional *PKD1* variant (Lyons et al., 2004). | | | | | | | |  |
