# Supplementary material for: Widespread genetic testing controls inherited polycystic kidney disease while avoiding inbreeding in cats
Source: BMC Genomics. 2026 Jun 18;27:716. doi: 10.1186/s12864-026-13048-4 (PMC13520345; doi:10.1186/s12864-026-13048-4)
Supplement: Supplementary file 2 [file 12864_2026_13048_MOESM2_ESM.docx]

Supplementary Table 2. Whole-genome and exome sequencing to detect novel variants associated with PKD

| Breed/group | Run/isolate | Sex | Disease/description |
| --- | --- | --- | --- |
| Ragamuffins | Case 1 | Female | PKD |
| Minuette | Case 2 | Female | PKD |
| Siberian | SRR11592781 | Female | PKD |
| Siberian | SRR23445356 | Male | PKD |
| Bengal | SRR23445358 | Female | PKD |
| Bengal | SRR25031651 | Male | PKD |
| Bengal | SRR25031655 | Female | PKD |
| Mixed breed | SRR25031663 | Female | PLD*/PKD |
| Himalayan | SRR25031672 | Male | PLD*/PKD |
| Abyssinian | SRR5055389 | Female | Progressive retinal atrophy (affected) |
| Abyssinian | SRR6997541 | Female | Amyloidosis (affected) |
| Abyssinian | SRR11392618 | Male | - |
| Abyssinian | SRR5373742 | Female | Amyloidosis (affected) |
| American Shorthair | takae | Female | - |
| American Shorthair | hutaba | Female | - |
| American Shorthair | F0174-01_Ams | Female | - |
| American Shorthair | senzu | Female | - |
| American Shorthair | SRR5051116 | Female | Niemann-Pick Type C (affected) |
| Bengal | SRR5051112 | Male | Normal |
| Bengal | SRR11392475 | Not collected | - |
| Bengal | SRR15512113 | Male | - |
| British Shorthair | rindou_Brt | Female | - |
| British Shorthair | ruri_Brt | Female | - |
| British Shorthair | jeni_Brt | Female | - |
| British Shorthair | ERR3486770 | Male | - |
| British Shorthair | ERR3486772 | Male | - |
| British Shorthair | ERR3486773 | Female | - |
| Exotic Shorthair | mochiko_Exo | Female | - |
| Exotic Shorthair | pito_Exo | Female | - |
| Exotic Shorthair | pugi_Exo | Female | - |
| Havana Brown | ERR3485138 | Female | - |
| Havana Brown | ERR3485139 | Male | - |
| Havana Brown | ERR3485140 | Female | - |
| Havana Brown | ERR3485141 | Male | - |
| Maine Coon | SRR11392457 | Not collected | - |
| Maine Coon | SRR11392458 | Not collected | - |
| Maine Coon | SRR11392459 | Not collected | - |
| Maine Coon | SRR11392460 | Not collected | - |
| Maine Coon | SRR11392461 | Not collected | - |
| Maine Coon | SRR11392470 | Not collected | - |
| Maine Coon | SRR11392505 | Male | - |
| Maine Coon | SRR11392506 | Male | - |
| Maine Coon | SRR11392510 | Female | - |
| Minuet | Minuet200218 | Male | - |
| Minuet | Minuet201225 | Female | - |
| Minuet | Minuet210504 | Male | - |
| Norwegian Forest | F0034 | Female | - |
| Norwegian Forest | mike_Nrw | Female | - |
| Norwegian Forest | mika_Nrw | Female | - |
| Oriental Shorthair | SRR5038369 | Female | Lymphoma (carrier) |
| Oriental Shorthair | SRR5038379 | Male | Lymphoma (carrier) |
| Oriental Shorthair | SRR5038384 | Female | Lymphoma (affected) |
| Persian | SRR9873363 | Not collected | Chediak-Higashi syndrome (affected) |
| Persian | SRR2224864 | Male | Progressive retinal atrophy (affected) |
| Persian | SRR13784147 | Male | - |
| Ragdoll | SRR5055396 | Female | - |
| Ragdoll | SRR5055399 | Female | - |
| Ragdoll | SRR8767304 | Unknown | Methemoglobinemia (affected) |
| Domestic Shorthair | SRR5055384 | Female |  |
| Domestic Shorthair | SRR5043265 | Female |  |
| Domestic Shorthair | SRR5040116 | Female | - |
| Domestic Shorthair | SRR5040113 | Male | - |
| Domestic Shorthair | SRR5040117 | Not collected | - |
| Domestic Shorthair | coco | Male | - |
| Domestic Shorthair | neko1 | Unknown | - |
| Domestic Shorthair | takeo | Female | - |
| Domestic Shorthair | Tsukushi_Velcat | Female | - |
| Domestic Shorthair | Daifuku | Male | - |
| Domestic Shorthair | Yamato | Male | - |
| Domestic Shorthair | Eru | Female | - |
| Domestic Shorthair | SRR5040118 | Female | Unknown |
| Domestic Shorthair | SRR5040111 | Not collected | Unknown |
| Domestic Shorthair | SRR5040107 | Female | - |
| Domestic Shorthair | SRR25031638 | Female | None |
| Domestic Shorthair | SRR25031639 | Male | None |
| Domestic Shorthair | SRR25031640 | Male | None |
| Domestic Shorthair | SRR25031641 | Male | None |
| Domestic Shorthair | SRR25031658 | Female | None |
| Domestic Shorthair | SRR5040126 | Female | - |
| Domestic Shorthair | ERR3595999 | Male | - |
| Domestic Shorthair | ERR5173701 | Female | - |
| Domestic Shorthair | ERR5173703 | Male | - |
| Domestic Shorthair | ERR6177054 | Male | - |
| Domestic Shorthair | SRR25054025 | Missing | 3-hydroxy-methalglutaconicaciduria (affected) |
| Domestic Shorthair | SRR25054042 | Female | - |
| Domestic Shorthair | SRR25054043 | Male | - |
| Domestic Shorthair | SRR25031035 | Missing | Osteopetrosis (affected) |
| Domestic Shorthair | ERR2497927 | Male | - |
| Domestic Shorthair | SRR5040110 | Female | - |
| Domestic Shorthair | SRR5040125 | Female | - |
| Domestic Shorthair | SRR25031038 | Missing | Mucopolysaccharidosis (affected) |
| Domestic Shorthair | SRR25031039 | Female | Hypercalcemia (affected) |
| Domestic Shorthair | SRR25031053 | Female | Cerebellar disease (affected) |
| Domestic Shorthair | SRR25031042 | Male | Diabetes (affected) |
| Domestic Shorthair | SRR25031063 | Female | Neuronal ceroid lipofuscinosis (affected) |
| Domestic Shorthair | SRR25031064 | Female | Neuromuscular disorder (affected) |
| Russian Blue | F0086-01 | Female | - |
| Russian Blue | F0131-01 | Female | - |
| Russian Blue | F0359_Rsb | Female | - |
| Scottish Fold | F0016-01_Sco | Female | - |
| Scottish Fold | F0306-01_Sco | Female | - |
| Scottish Fold | F0298-02_Sco | Female | - |
| Siamese | ERR3485781 | Male | - |
| Siamese | SRR11392454 | Not collected | - |
| Siamese | SRR11392455 | Not collected | - |
| Siamese | SRR5363128 | Male | - |

*PLD: polycystic liver disease.
