# Supplementary material for: Widespread genetic testing controls inherited polycystic kidney disease while avoiding inbreeding in cats
Source: BMC Genomics. 2026 Jun 18;27:716. doi: 10.1186/s12864-026-13048-4 (PMC13520345; doi:10.1186/s12864-026-13048-4)
Supplement: Supplementary file 3 [file 12864_2026_13048_MOESM4_ESM.docx]

Supplementary Table 6. Comparison of observed heterozygosity.

| Breeds | Group | Heterozygosity | CI  2.5% | CI  97.5% |
| --- | --- | --- | --- | --- |
| Scottish Fold | Hetero 2019 | 0.29 | 0.292 | 0.296 |
|  | Wild 2019 | 0.30 | 0.296 | 0.299 |
|  | Hetero 2022 | 0.31 | 0.312 | 0.315 |
|  | Wild 2022 | 0.31 | 0.309 | 0.313 |
| Persian | Hetero 2019 | 0.28 | 0.278 | 0.282 |
|  | Wild 2019 | 0.28 | 0.278 | 0.282 |
|  | Hetero 2022 | 0.28 | 0.277 | 0.281 |
|  | Wild 2022 | 0.28 | 0.278 | 0.282 |
