# Supplementary material for: Widespread genetic testing controls inherited polycystic kidney disease while avoiding inbreeding in cats
Source: BMC Genomics. 2026 Jun 18;27:716. doi: 10.1186/s12864-026-13048-4 (PMC13520345; doi:10.1186/s12864-026-13048-4)
Supplement: Supplementary file 4 [file 12864_2026_13048_MOESM5_ESM.docx]

Supplementary Table 5. Logistic regression statistics for fluctuations in *PKD1* variant frequency

| Breed | Estimate | Std. Error | z value | Pr (>\|z\|) |  |
| --- | --- | --- | --- | --- | --- |
| Scottish Fold | -0.263 | 0.052 | -5.057 | 4.E-07 | ** |
| American Shorthair | -0.254 | 0.097 | -2.625 | 0.009 | * |
| Munchkin | -0.030 | 0.070 | -0.423 | 0.672 |  |
| British Shorthair | -0.023 | 0.127 | -0.181 | 0.857 |  |
| Minuet | -0.154 | 0.079 | -1.945 | 0.052 |  |
| Persian | -0.248 | 0.077 | -3.228 | 0.001 | ** |
| Ragamuffin | -0.316 | 0.104 | -3.022 | 0.003 | ** |
| Exotic Shorthair | -0.218 | 0.083 | -2.620 | 0.009 | * |
| No analyses performed for Ragdolls because no Hetero was detected in all years. ** and * indicate significant and suggestive level with Bonferroni correction (0.05/8 and 0.1/8). | | | | | |
|  | | | | | |
