# Supplementary material for: Widespread genetic testing controls inherited polycystic kidney disease while avoiding inbreeding in cats
Source: BMC Genomics. 2026 Jun 18;27:716. doi: 10.1186/s12864-026-13048-4 (PMC13520345; doi:10.1186/s12864-026-13048-4)
Supplement: Supplementary file 5 [file 12864_2026_13048_MOESM6_ESM.docx]

Supplementary Table 4. Number of cats used for allele frequency analysis of conventional *PKD1* variants

| Breeds | Birth Year | Hetero | Wild | All | Ratio | Δratio (vs 2019) |
| --- | --- | --- | --- | --- | --- | --- |
| Scottish Fold (n=16,451) | 2019 | 130 | 3,998 | 4,128 | 3.15% | - |
|  | 2020 | 97 | 4,345 | 4,442 | 2.18% | -30.79% |
|  | 2021 | 77 | 4,594 | 4,671 | 1.65% | -47.62% |
|  | 2022 | 51 | 3,159 | 3,210 | 1.59% | -49.52% |
| American Shorthair (n=10,242) | 2019 | 39 | 2,618 | 2,657 | 1.47% | - |
|  | 2020 | 28 | 2,867 | 2,895 | 0.97% | -34.01% |
|  | 2021 | 24 | 2,829 | 2,853 | 0.84% | -42.86% |
|  | 2022 | 13 | 1,824 | 1,837 | 0.71% | -51.70% |
| Munchkin (n=10,132) | 2019 | 46 | 2,428 | 2,474 | 1.86% | - |
|  | 2020 | 41 | 2,706 | 2,747 | 1.49% | -19.89% |
|  | 2021 | 71 | 2,789 | 2,860 | 2.48% | 33.33% |
|  | 2022 | 25 | 2,026 | 2,051 | 1.22% | -34.41% |
| British Shorthair (n=6,477) | 2019 | 16 | 1,597 | 1,613 | 0.99% | - |
|  | 2020 | 14 | 1,757 | 1,771 | 0.79% | -20.20% |
|  | 2021 | 10 | 1,754 | 1,764 | 0.57% | -42.42% |
|  | 2022 | 14 | 1,315 | 1,329 | 1.05% | 6.06% |
| Minuet (n=4,540) | 2019 | 42 | 705 | 747 | 5.62% | - |
|  | 2020 | 29 | 1,172 | 1,201 | 2.41% | -57.12% |
|  | 2021 | 49 | 1,416 | 1,465 | 3.34% | -40.57% |
|  | 2022 | 35 | 1,092 | 1,127 | 3.11% | -44.66% |
| Ragamuffin (n=3,928) | 2019 | 21 | 559 | 580 | 3.62% | - |
|  | 2020 | 27 | 963 | 990 | 2.73% | -24.59% |
|  | 2021 | 22 | 1,228 | 1,250 | 1.76% | -51.38% |
|  | 2022 | 17 | 1,091 | 1,108 | 1.53% | -57.73% |
| Persian (n=3,596) | 2019 | 59 | 819 | 878 | 6.72% | - |
|  | 2020 | 43 | 917 | 960 | 4.48% | -33.33% |
|  | 2021 | 25 | 954 | 979 | 2.55% | -62.05% |
|  | 2022 | 32 | 747 | 779 | 4.11% | -38.84% |
| Ragdoll (n=3,150) | 2019 | 0 | 565 | 565 | 0.00% | - |
|  | 2020 | 0 | 705 | 705 | 0.00% | 0.00% |
|  | 2021 | 0 | 822 | 822 | 0.00% | 0.00% |
|  | 2022 | 0 | 1,058 | 1,058 | 0.00% | 0.00% |
| Exotic Shorthair (n=2,381) | 2019 | 42 | 560 | 602 | 6.98% | - |
|  | 2020 | 45 | 549 | 594 | 7.58% | 8.60% |
|  | 2021 | 27 | 670 | 697 | 3.87% | -44.56% |
|  | 2022 | 22 | 466 | 488 | 4.51% | -35.39% |
| Himalayan (n=542) | 2019 | 19 | 116 | 135 | 14.07% | - |
|  | 2020 | 9 | 131 | 140 | 6.43% | -54.30% |
|  | 2021 | 9 | 172 | 181 | 4.97% | -64.68% |
|  | 2022 | 8 | 78 | 86 | 9.30% | -33.90% |
| Siberian (n=233) | 2019 | 3 | 40 | 43 | 6.98% | - |
|  | 2020 | 5 | 47 | 52 | 9.62% | 37.82% |
|  | 2021 | 4 | 79 | 83 | 4.82% | -30.95% |
|  | 2022 | 1 | 54 | 55 | 1.82% | -73.93% |
| Maine Coon (n=158) | 2019 | 0 | 25 | 25 | 0.00% | - |
|  | 2020 | 0 | 67 | 67 | 0.00% | 0.00% |
|  | 2021 | 0 | 44 | 44 | 0.00% | 0.00% |
|  | 2022 | 0 | 22 | 22 | 0.00% | 0.00% |
| Bengal (n=88) | 2019 | 0 | 17 | 17 | 0.00% | - |
|  | 2020 | 0 | 26 | 26 | 0.00% | 0.00% |
|  | 2021 | 0 | 29 | 29 | 0.00% | 0.00% |
|  | 2022 | 0 | 16 | 16 | 0.00% | 0.00% |
| Russian Blue (n=50) | 2019 | 0 | 13 | 13 | 0.00% | - |
|  | 2020 | 0 | 10 | 10 | 0.00% | 0.00% |
|  | 2021 | 0 | 15 | 15 | 0.00% | 0.00% |
|  | 2022 | 0 | 12 | 12 | 0.00% | 0.00% |
| All breeds (n=61,968) | 2019 | 417 | 14,060 | 14,477 | 2.88% | - |
|  | 2020 | 338 | 16,262 | 16,600 | 2.04% | -29.31% |
|  | 2021 | 318 | 17,395 | 17,713 | 1.80% | -37.67% |
|  | 2022 | 218 | 12,960 | 13,178 | 1.65% | -42.57% |

* *PKD1* variant homozygosity is embryonically lethal (refer to the main text for further details).
