# Supplementary material for: Widespread genetic testing controls inherited polycystic kidney disease while avoiding inbreeding in cats
Source: BMC Genomics. 2026 Jun 18;27:716. doi: 10.1186/s12864-026-13048-4 (PMC13520345; doi:10.1186/s12864-026-13048-4)
Supplement: Supplementary file 6 [file 12864_2026_13048_MOESM7_ESM.docx]

**Supplementary Table 1.** Number of cats insured for >10 years and renal cyst status

| Breed | Sex | Cyst-positive | Non-positive | Total | Ratio |
| --- | --- | --- | --- | --- | --- |
| Scottish Fold | Female | 6 | 1,738 | 1,744 | 0.0034 |
|  | Male | 4 | 2,101 | 2,105 | 0.0019 |
|  | Total | 10 | 3839 | 3849 | 0.0026 |
| Munchkin | Female | 1 | 602 | 603 | 0.0017 |
|  | Male | 0 | 680 | 680 | 0 |
|  | Total | 1 | 1282 | 1283 | 0.0008 |
| American Shorthair | Female | 5 | 1,205 | 1,210 | 0.0041 |
|  | Male | 1 | 1,487 | 1,488 | 0.0007 |
|  | Total | 6 | 2692 | 2698 | 0.0022 |
| Persian | Female | 3 | 386 | 389 | 0.0077 |
|  | Male | 1 | 434 | 435 | 0.0023 |
|  | Total | 4 | 820 | 824 | 0.0049 |
| All breeds |  | 21 | 8,633 | 8,654 | 0.0024 |
